# Preparation of Solid Lipid Nanoparticles of Cinnamaldehyde and Determination of Sustained Release Capacity

**DOI:** 10.3390/nano12244460

**Published:** 2022-12-15

**Authors:** Jiajia Chen, Shangjian Li, Qinhua Zheng, Xiaolin Feng, Weijian Tan, Kexin Feng, Yuntong Liu, Wenzhong Hu

**Affiliations:** 1 School of Pharmacy and Food Science, Zhuhai College of Science and Technology, Zhuhai 519040, China; 2 College of Life Science, Jilin University, Changchun 130012, China

**Keywords:** cinnamaldehyde, solid lipid nanoparticles, sustained release

## Abstract

Natural plant essential oils cannot be applied on a large scale due to their high volatility, easy deactivation, etc. This study provides a new method to prepare a long-lasting, slow-release essential oil product by taking advantage of solid lipid nanoparticles, which will provide a scientific guideline for the future essential oil industry. In this article, solid lipid cinnamaldehyde nanoparticles were prepared using an ultrahigh-pressure homogenization method. SLN-CA with a particle size of 74 ± 5 nm, PDI of 0.153 ± 0.032, and zeta potential of −44.36 ± 2.2 mV was screened using an additional amount of cinnamaldehyde, the ratio of oil phase components, and the homogenization pressure and number of times as factors. Differential thermal analysis and spectroscopy demonstrated that cinnamaldehyde was successfully encapsulated inside the nanoparticles. The change in particle size of nanoparticles under different conditions and times was used as an indicator of stability. The stability of the finished nanoparticles was evaluated. The retention and slow-release ability of cinnamaldehyde were investigated using the concentration of cinnamaldehyde in nanoparticles as an indicator. The results showed that after 15 days, SLN-CA retained 52.36% of the concentration from 15 days prior. The bacterial inhibition test shows that SLN-CA can inhibit bacteria

## 1. Introduction

**Figure 1:**
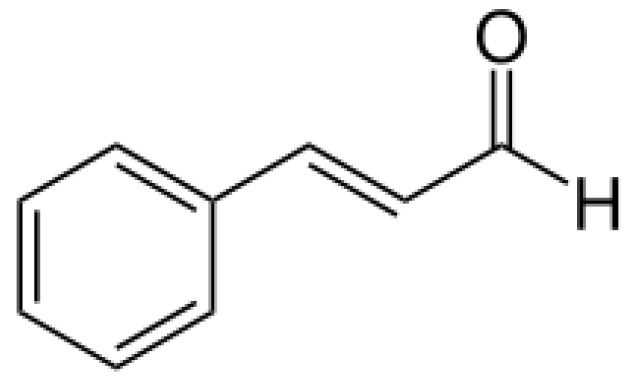
Chemical structure of cinnamaldehyde.

Infestation by pathogenic microorganisms is one of the main factors causing spoilage of fruits and vegetables [1]. Cinnamaldehyde is a natural antibacterial agent with good antibacterial effect, Inhibits the growth of many species of bacteria such as *Escherichia coli*, *Salmonella*, *Bacillus subtilis*, *Pseudomonas aeruginosa*, *Aspergillus*, and *Fusarium acnes* [2,3,4]. The chemical structure of cinnamaldehyde is shown in Figure 1. Cinnamaldehyde has a pungent cinnamon aroma; therefore, its direct addition to food can affect the organoleptic properties [5]. It has poor stability and can volatilize with water vapor, and is easily oxidized to cinnamic acid in air. Cinnamic aldehyde is soluble in alcohols, ethers, chloroform and other organic solvents. The solubility of cinnamaldehyde in water is very low—1 g/L at 20 °C. Due to various defects, it is difficult to apply cinnamaldehyde in the fruit and vegetable preservation industry on a large scale.

**Figure 2:**
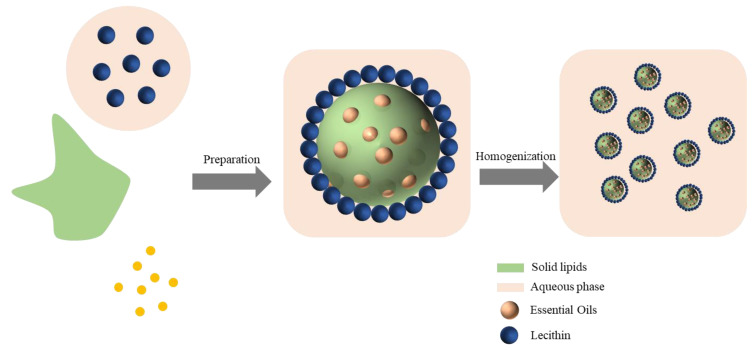
Schematic diagram of solid-lipid-nanoparticle-embedded active substance and preparation.

Solid lipid nanoparticles (SLNs) are among the nanoscale drug carriers developed in the 1980s and 1990s. SLNs have a low toxicity, and a solid and highly bioavailable lipid as a liposomal wall at room temperature, which provides in vivo slow release of long-acting drugs, improved drug efficacy, reduced toxicity before reaching the site of action, improved bioavailability of drugs, protection of bioactive compounds and enhanced physiological compatibility [6,7,8,9]. Therefore, encapsulating cinnamaldehyde in solid lipid nanoparticles protects its antibacterial activity and prolongs its duration of action. Schematic diagram of solid-lipid-nanoparticle-embedded active substance and preparation (Figure 2).

The aim of this experiment is to investigate a new efficient and environmentally friendly natural bacterial inhibitor. SLN-cinnamaldehyde (SLN-CA) was prepared by high-pressure microinjection method using monostearin as a wall material and cinnamaldehyde as an encapsulating material characterized and identified by its slow-release ability.

## 2. Materials and Methods

### 2.1. Materials and Reagents

Cinnamaldehyde (CA) (AR, 95%, CAS: 104-55-2, Shanghai Yuan Ye Bio-Technology Co., Ltd., Shanghai, China). Poloxam-188(BR, CAS: 9003-11-6, Shanghai Yuan Ye Bio-Technology Co., Ltd., Shanghai, China). Monostearin (MG) (AR, CAS: 123-94-4, Shanghai Yuan Ye Bio-Technology Co., Ltd., Shanghai, China). Soy lecithin (Sheng Qing Bio, Shanghai, China). Tween-80 (T-80) (PC, CAS: 9005-65-6, Shanghai Yuan Ye Bio-Technology Co., Ltd., Shanghai, China). Phosphotungstic acid negative staining solution (RT, 2%, BIOLAB, Beijing, China). Phosphate buffer saline (PBS) (BR, Shanghai Yuan Ye Bio-Technology Co., Ltd., Shanghai, China). Ethanol absolute (AR, ≥99.7%, CAS: 64-17-5, Sinopharm Chemical Reagent Co., Ltd., Sinopharm Chemical Reagent Co., Ltd.). *Escherichia coli* (CGMCC). *Staphylococcus aureus* (GDMCC). *Creeping Rhizopus* (GDMCC). *Aspergillus niger* (GDMCC).

### 2.2. Instruments and Equipmen

Ultra-high-pressure homogenizer (Nano DeBEE, BEE International, South Easton, MA 02375, USA). LS Particle Size Analyzer (LS 13 320, Beckman Coulter, Brea, CA, USA). Scanning electron microscope (TESCAN MIRA, TESCAN, Brno, Czech Republic). UV spectrophotometer (Shimadzu, UV-2600i, Kyoto, Japan). Frozen centrifuges (1248R, genecompany, HK, China). Transmission electron microscope (HT7700, Hitachi, Tokyo, Japan). Differential scanning calorimeter (DSC 214 Polyma, netzsch, Bavaria, Germany).

### 2.3. Preparation and Characterization of SLN-CA

#### 2.3.1. Preparation of SLN-CA

Cinnamic aldehyde solid lipid nanoparticles (SLN-CA) were prepared using a high-pressure microfluidic method [10,11,12]. In total, 1.5 g of Poloxamer 188 and 1.5 g of Lecithin High Potency were added to 100 mL of ultrapure water to form the aqueous phase and stirred on a magnetic stirrer at 65 °C. A total amount of 2 g of T-80 and MG was placed on a magnetic stirrer at 65 °C to melt and mix thoroughly. A total of 1 mL essential oil was added to the mixture and stirred well. The aqueous phase was added to the oil phase at a rate of 6.6 mL per minute and timed for 15 min. Finally, the mixture was homogenized under high pressure with a micro jet homogenizer and then chilled in a refrigerator for 1 h to obtain SLN-CA.

#### 2.3.2. Process and Prescription Optimization

Different conditions were changed during preparation, such as the working pressure of the homogenizer (5000 psi, 10,000 psi, 15,000 psi, 20,000 psi, 25,000 psi), the number of homogenizations (0, 1, 2, 3, 4, 5) and the amount of Tween-80: monostearin in the prescription (0:2, 0.3:1.7, 0.7:1.3, 1:1, 1.3:0.7, 1.7:0.3), the amount of CA (1 mL, 2 mL, 3 mL, 4 mL, 5 mL, 6 mL); then, the particle size and encapsulation rate were used as indicators to determine the best process and formulation.

#### 2.3.3. Particle Size Distribution

An appropriate amount of SLN-CA suspension was measured at room temperature (25 °C), and using the liquid analysis module of the laser diffraction particle size analyzer, the test temperature was set to 25 °C at room temperature, the scattering angle was 90°, the optical model was selected as: psl.rf780d [10], and the measurements were repeated three times, with 100 runs for each measurement, respectively. Photon correlation spectroscopy (PCS) was performed to determine the average particle size of SLN-CA.

#### 2.3.4. Zeta-Potential

An appropriate amount of SLN-CA suspension was measured at room temperature (25 °C), and the zeta potential of SLN-CA was determined by electrophoretic light scattering (DLS) using a NanoPlus HD nanoparticle size and zeta potential meter with the test temperature set at room temperature (25 °C) and the scattering angle set to 90°.

#### 2.3.5. Polydispersity

The polydispersity coefficients of SLN-CA were measured by dynamic light scattering (DLS) at room temperature (25 °C) using a NanoPlus HD nanoparticle size and zeta potential meter, with the test temperature set at 25 °C and the scattering angle at 90°.

#### 2.3.6. Absorption Spectra

A specific concentration of CA, SLN, and SLN-CA samples was taken. SLN-CA removes free CA by ultrafiltration to prevent it from interfering with subsequent analysis. CA, SLN, and SLN-CA were scanned with the spectral scan function of the UV spectrophotometer, and the curves in the absorbance range of 0.2–0.8 were selected for analysis. Each experimental group was repeated three times.

#### 2.3.7. Encapsulation Percentage

The concentration of CA in SLN-CA was measured using a UV spectrophotometer, and the percentage of encapsulation (EN) of SLN-CA was determined. Three identical batches of SLN-CA 1.5 mL were accurately pipetted into 3 mL ultrafiltration tubes [11], centrifuged at 6000 rpm for 20 min, and the upper and lower layers were removed separately in different brown frosted bottles. The liquid was diluted with anhydrous ethanol to a suitable concentration and the absorbance was measured at 285 nm using a UV spectrophotometer. Another 1.5 mL SLN-CA suspension was diluted with anhydrous ethanol to form a suitable concentration. The absorbance was measured at 285 nm using a UV spectrophotometer, and the total essential oil concentration was calculated according to the regression equation of the standard curve. Standard curve equation: C = (A − 0.0154)/391.82, R^2^ = 0.9993 (n = 6).

$$EN\left(\%\right)=\left(1-\frac{C_{free}\times V_{free}}{C_{Total}\times V_{Total}}\right)\times 100\%$$

In the formula: $C_{free}$ (Concentration of free cinnamaldehyde); $C_{Total}$ (Concentration of cinnamaldehyde in the suspension); $V_{free}$ (Volume of the lower solution of the ultrafiltration tube); $V_{Total}$ (total concentration volume of SLN-CA suspension).

#### 2.3.8. Nanoparticle Morphology

The shape and appearance were observed by scanning electron microscopy (SEM) and transmission electron microscopy (TEM). Both scanning electron and transmission electron microscopy were used to observe the shape and appearance. The samples were taken out, and a tiny amount of SLN-CA was added dropwise on top of the SEM particular silicon wafer and TEM special copper mesh sieve. The silicon wafer was put into a 45 °C oven to dry for 24 h, and the copper mesh was negatively stained with phosphotungstic acid negative stain solution and put into an oven at 45 °C to dry. The dried samples were observed using the instruments mentioned.

#### 2.3.9. Differential Scanning Calorimetry

Differential scanning calorimetry (DSC) is often used to study the crystallization patterns and polymorphic behavior of the formulation components of NLCs and SLNs [12]. Approximately 10 mg of SLN-CA lyophilized powder and a physical mixture of SLN and essential oil were weighed in an aluminium crucible under a dynamic nitrogen atmosphere, with an empty crucible as a control. Manual calibration parameters. The scanning temperature range is 0–100 °C, and the rate is 10 °C/min. The starting temperature (To), final temperature (Tc), and peak temperature (Tp) of the analysis were recorded by the STARe thermal analysis software. The enthalpy of melting (ΔH) of SLN-CA was calculated from the area between the thermal spectrum and the baseline under the peak.

#### 2.3.10. Fourier Transform Infrared Spectroscopy (FT-IR)

FT-IR is commonly used to verify and detect whether the inclusion complex interacts with the enclosed complex [13,14]. Weigh the appropriate amount of SLN-CA lyophilized powder at room temperature (25 °C) and mix the sample, lyophilized SLN, and physical mixture with KBr by grinding thoroughly. CA uses the liquid preparation method to prepare samples and replaces the liquid module on the instrument for testing. The infrared spectrometer operating conditions were set at a spectral range of 500–4000 cm^−1^, 4 cm^−1^ spectral resolution, in the mid-infrared region inside the sample [15].

#### 2.3.11. Retardation and Retention

SLN-CA was left with the same concentration of CA at room temperature (25 °C) for 15 days; next, 1 mL SLN-CA was added to 9 mL of anhydrous ethanol every 3 days, and the wall was broken by ultrasonication for 1 min, the supernatant was taken after centrifugation, the absorbance was detected at 285 nm using a UV spectrophotometer, and the concentration of cinnamaldehyde was calculated according to the standard curve of 3.1.4. CA was taken directly from the sample under the same conditions. The concentration was measured under the same conditions. Each group was repeated three times. The formula calculates the retention rate:

$$Retention\;rate\;\left(\%\right)=\frac{C_{n}}{C}$$

In the formula is the concentration on day *n*, and *C* is the initial concentration.

#### 2.3.12. Stability

(1) Changes in the appearance of SLN-CA before and after 6 d storage

SLN-CA was stored in colourimetric tubes at room temperature (25 °C) for six days to observe whether there was stratification before and after, and each group of tests was repeated three times.

(2) Changes in particle size of SLN-CA before and after storage for 6 d

SLN-CA was stored at room temperature (25 °C) for six days to measure the particle size. Each group of experiments was repeated three times.

(3) Effect of different pH values on the particle size of SLN-CA

SLN-CA was adjusted to pH 3.17, 4.17, 5.17, 6.17 and 7.17 with 0.1 mol/mL NaOH or 1% phosphoric acid, and the particle size of SLN-CA was measured at different pH values.

(4) Effect of different pH values on the particle size of SLN-CA in storage for 6 days

The pH of SLN-CA was adjusted to 3.17, 4.17, 5.17, 6.17, 7.17 with 0.1 mol/mL NaOH or 1% phosphoric acid, and the particle size was measured by storing SLN-CA at room temperature (25 °C) for six days.

(5) Effect of different temperatures on the particle size of SLN-CA stored for 6 days

SLN-CA was stored at 35 °C and 55 °C for six days to measure the particle size. Each group of tests was repeated three times.

### 2.4. Inhibition of Bacteria and Fungi

Determination of the inhibition effect of SLN-CA was carried out on two foodborne pathogenic microorganisms and three fruit and vegetable spoilage fungi using the perforation method [16]. Each plate was coated with 25 mL of medium (TSA for bacteria and PDA for fungi) [17,18]. SLN-CA was filtered and decontaminated using 0.22 μm PTFE needle-type microporous membrane filters and stored at room temperature. In total, 100 μL of bacterial broth with a concentration of 10^8^ cfu/mL was taken and incubated at 37 °C for 24 h or eluted at 28 °C with a concentration of 10^8^ cfu/mL. The plate was coated with the spore solution of 10^8^ cfu/mL and punched on the solidified plate with a 6 mm gel punch, then 90 μL of SLN-CA was added to the wells, sealed with the plate membrane and incubated at 37 °C (24 h for bacteria and 72 h for fungi), and the size of the inhibition circle was measured and photographed using the microbial colony analysis system. The criteria for determining the inhibition effect of SLN-CA on foodborne pathogenic microorganisms and pathogenic fungi of fruits and vegetables are as follows.

(1) The diameter of the inhibition circle size is ≥20 mm. The inhibition effect is susceptible.
(2) The diameter of the inhibition circle size in the middle of 12–20 mm inhibition for moderate sensitivity.
(3) The diameter of the inhibition circle size of ≤12 mm inhibition for low sensitivity.

The size of the inhibition circle of each bacteria test was repeated three times.

## 3. Results and Discussion

### 3.1. Preparation and Characterization of SLN-CA

#### 3.1.1. Preparation of SLN-CA

Based on the results of previous pre-experiments, the initial conditions were set as follows: homogenization pressure of 10,000 psi, homogenization once, T80 to MG ratio of 1:1, and 1 mL dose of essential oil.

**Figure 3:**
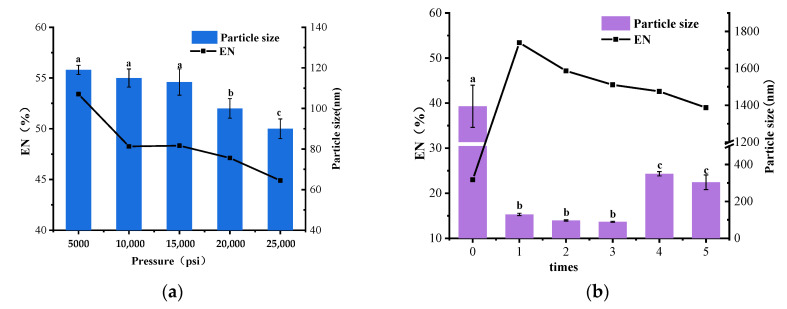
Effect of different homogenization pressures on the particle size and encapsulation rate of SLN-CA during preparation in a single-factor experiment (**a**); effect of different homogenization times on the particle size and encapsulation rate of SLN-CA during preparation in a single-factor experiment (**b**); error bars indicate the standard deviation of the mean (three replicates); different letters a–c in the figure indicate significant differences in particle size results between treatments based on Duncan’s multiple range test (*p* < 0.05).

Optimization of the pressure of the homogenizer was essential in the Preparation of SLN-CA, and the single-factor experiments were carried out at 5000 psi, 10,000 psi, 15,000 psi, 20,000 psi, and 25,000 psi. The results showed that the higher the pressure of the ultra-high-pressure homogenizer, the smaller the particle size of SLN-CA; however, the encapsulation rate also decreased (Figure 3a). Other factors were kept constant, and the number of homogenization times was changed to 1, 2, 3, 4 and 5 times. The results showed that the sample without homogenization had a huge dispersed particle size, and the encapsulation rate was only 23%. The particle size decreased and then increased as the number of times increased. After one round of homogenization, the encapsulation rate of SLN-CA also decreased more and more with the increase in pressure (Figure 3b).

The encapsulation rate decreases with increasing homogenization pressure and the number of times. It is because Cinnamaldehyde is a volatile material [19]. The temperature of the SLN-CA suspension rises with the homogenization pressure when it passes through the high-pressure microjet homogenization. A higher temperature accelerates the volatilization of essential oils and decreases the encapsulation rate of SLN-CA. 

Unhomogenized SLN-CA has huge particle size and dispersion. The main reason is the lack of homogenization under high pressure to disperse the suspension and shear the nanoparticles sufficiently. The low encapsulation rate is due to the agglomeration phenomenon of CA, which makes the nanoparticles dispersed at the interface between the aqueous and oil phases cannot encapsulate the essential oil of cinnamaldehyde well or after encapsulation. The stability is poor due to the large particle size, and the essential oil leaks out when the nanoparticles break. These reasons combined lead to a low encapsulation rate of SLN-CA without homogenization treatment.

The particle size is reduced because the nanoparticles are strongly sheared by high pressure. The higher the pressure, the higher the shear force, and the smaller the particle size [20].

**Figure 4:**
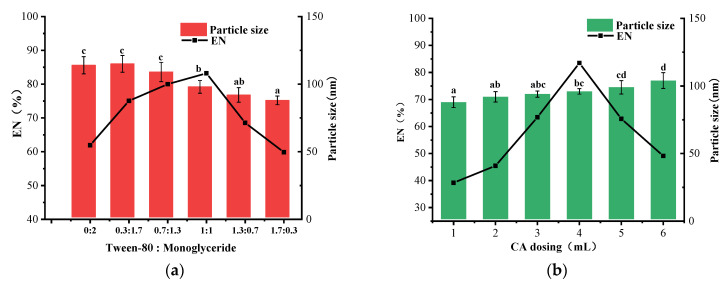
Effect of different ratios of T-80 to MG on the particle size and encapsulation rate of SLN-CA during preparation in a single-factor experiment (**a**); effect of different CA dosing on the particle size and encapsulation rate of SLN-CA during preparation in a single-factor experiment (**b**); error bars indicate the standard deviation of the mean (three replicates); different letters a–c in the figure indicate significant differences in particle size results between treatments based on Duncan’s multiple range test (*p* < 0.05).

Fixing other factors and varying the ratio of T-80 to MG (0:2, 0.3:1.7, 0.7:1.3, 1:1, 1.3:0.7, 1.7:0.3), the results showed that the highest encapsulation rate was achieved when the ratio of T-80 to MG was 1:1 in the oil-based formula. As the ratio of MG increases, the particle size also increases. (Figure 4a). T-80 can fully make the spheres formed by monostearin acid maintain the spherical structure better during the moving process and improve the structure and stability of the lipid wall material. Additionally, a high percentage of monostearin glycerides increases the suspension’s viscosity and resistance to shear forces, increasing the particle size [21].

However, a high percentage of T-80 decreases the concentration of MG in the suspension, reducing the number of lipid globules and, thus, the encapsulation rate of SLN-CA. From the particle size point of view, although the high percentage of T-80 can reduce the particle size of nanoparticles, it will also significantly reduce the encapsulation rate of SLN-CA. Therefore, from the perspective of the production application, the T-80:MG ratio is 1:1, which has a smaller particle size and a higher encapsulation rate.

Fixing other factors and varying the amount of essential oil (1, 2, 3, 4, 5 mL), the results showed that the particle size becomes more prominent as the dosage increases. The encapsulation takes the lead in increasing and then decreasing (Figure 4b) because a shortage of CA can lead to the appearance of empty nanoparticles, and too large can lead to an oversupply of nanoparticles.

In summary, according to the results of the experiments and process experiments for screening the best prescription, the homogenization pressure of 15,000 psi, the number of homogenizations of 2 times, the Tween-80: glycerol monostearate of 1:1, and the amount of essential oil of 4 mL were selected as conditions for the preparation of SLN-CA for further characterization and detection.

#### 3.1.2. Particle Size, Zeta-Potential and Polydispersity Coefficient

**Figure 5:**
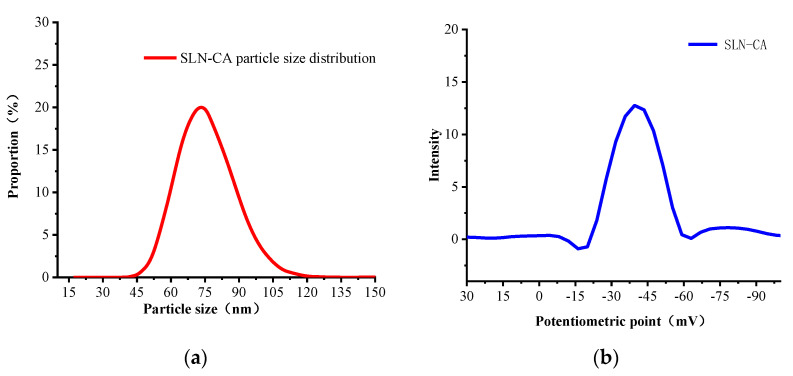
Results of particle size, zeta potential and polydispersity coefficient: (**a**) particle size and distribution; (**b**) zeta potential distribution.

Particle size, PDI is often used to evaluate the dispersion of a system, and PDI values are usually low for a homogeneous system (0.001–0.290) [22]. Zeta-potential is an important indicator to determine the physical stability of nanoparticles, which indirectly surfaces the physical stability of nanoparticles. It is a measurement of the particle surface charge and an indirect measurement of the diffusion layer thickness. The level of zeta potential is related to the tendency of aggregation or flocculation, and a considerable zeta potential indicates good stability of the colloid of the nanoformulation. The particle size of SLN-CA prepared in this study was 74 nm, with a small particle size, zeta-potential of −44.46 mV, and polydispersity coefficient of 0.15 (Table 1). The particle size results showed that the size of SLN-CA was concentrated around 74 nm and distributed to both sides according to the particle size distribution pattern (Figure 5a). The zeta-potential is −44.36 mV (Figure 5b), indicating that SLN-CA has good stability [23,24].

#### 3.1.3. UV Absorption Spectrum

**Figure 6:**
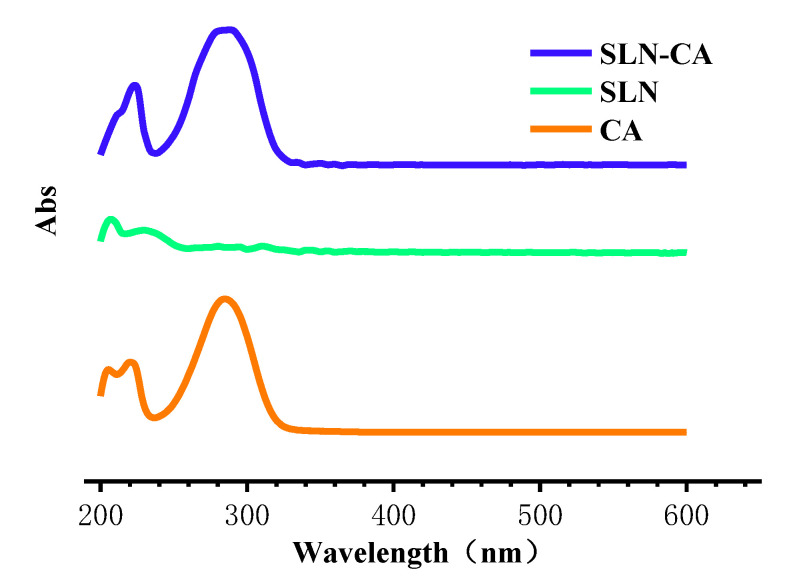
CA, SLN and SLN-CA of UV absorption spectrum.

UV absorption spectroscopy is widely used in the analysis of various herbal medicines [25]. We can see that CA has the maximum absorption peak at 285 nm, and SLN-CA has the maximum absorption peak at 285 nm, which is consistent with that of CA. SLN has no UV absorption at 285 nm (Figure 6). Since SLN-CA has been ultrafiltered to remove free CA interference before detection, it is clear from the results of the scanning UV spectrum that SLN-CA still has a maximum absorption at 285 nm; therefore, SLN-CA has successfully encapsulated CA into SLN.

#### 3.1.4. Standard Curves

**Figure 7:**
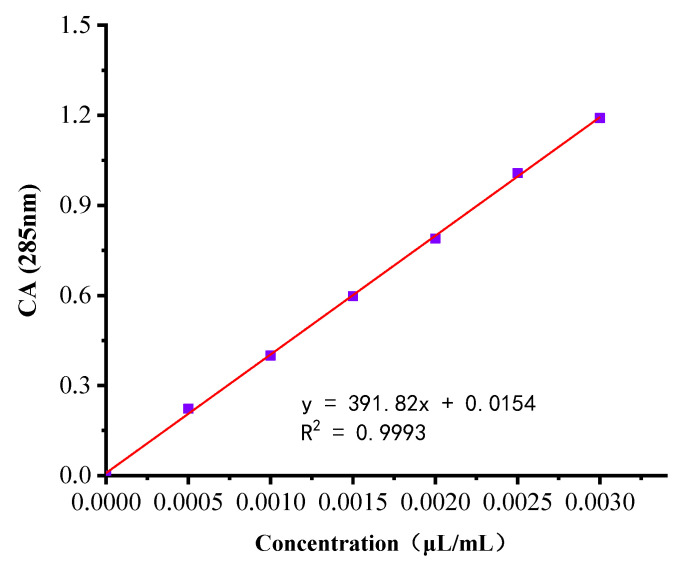
CA absorbance at 220 nm vs. concentration standard curve.

The relationship between the absorbance and concentration of cinnamaldehyde is shown at 285 nm as a standard curve (Figure 7). Since the fit of the fitted equation equals 0.9993, the linear regression equation is an excellent fit for the observed values. The standard equation can calculate the concentration very well when the CA concentration is between 0.0005 μL/mL and 0.003 μL/mL. The concentration equation was $C=\frac{A-0.0154}{391.82}\;$.

#### 3.1.5. FT-IR

**Figure 8:**
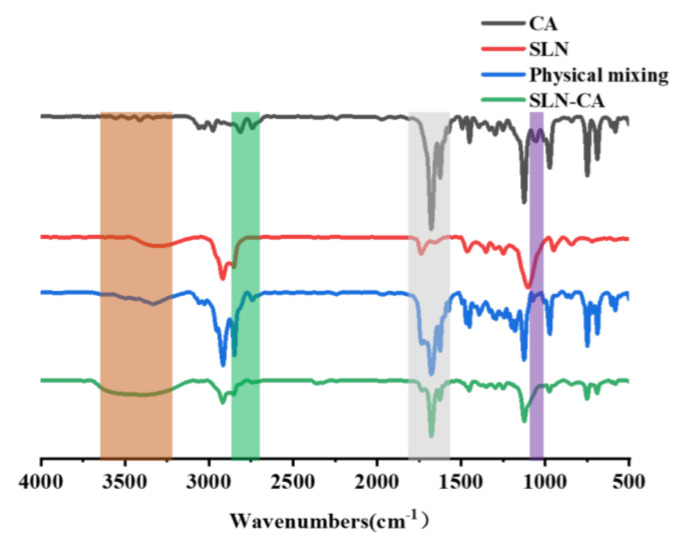
CA, SLN, physical mixture, FT-IR spectrum of SLN-CA.

The FT-IR results show that the CA patterns at 2861 cm^−1^ and 2742 cm^−1^ are generated by the C-H stretching vibration of the aldehyde group, and the C=O has a stretching vibration at 1678 cm^−1^ (Figure 8). The physical mixture profile appears to be a superposition of the two, showing the characteristic absorption peak of CA and the characteristic absorption of SLN. It is proven that in the physical mixture, there is no interaction between the components of CA and SLN, but simply mixing [26]. In the spectrum of SLN-CA, the C-H of the aldehyde group of CA 2861 cm^−1^ and 2742 cm^−1^ disappeared, and the peaks at 3100–3500 cm^−1^ became broad and significant, indicating some binding interaction between CA and the nanoparticles. On the other hand, there are no new absorption peaks in the whole spectrum (Figure 8) [14].

#### 3.1.6. SEM and TEM

**Figure 9:**
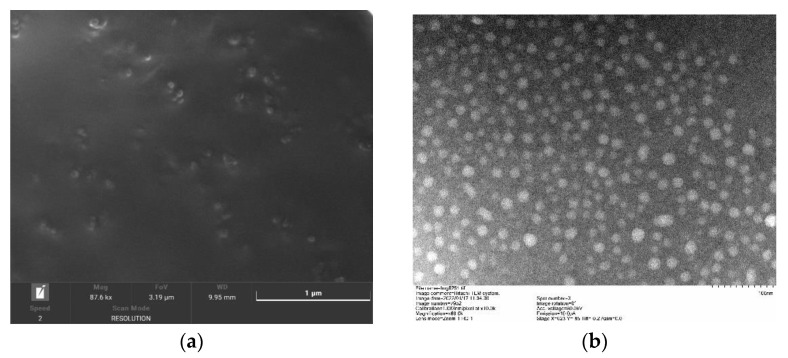
SLN-CA scanning electron microscope morphology (**a**), SLN-CA transmission electron microscope morphology (**b**).

Figure 9a is the result of SLN-CA observed at 87.6 k magnification under SEM; the nanoparticle interior is brighter, surrounded by a darker, rounded, and complete exterior. SLN-CA was observed under TEM at 60 k magnification. The sample is stained by the negative staining method; thus, the background is darker, with the sample showing high levels of brightness. The result shows that the nanoparticles are uniformly dispersed and uniform in size as rounded particles [27] (Figure 9b). The SEM and TEM observations show that the prepared SLN-CA suspensions are uniformly dispersed, and the nanoparticle structure is intact as spheres, with particle sizes ranging from about 15 to 30 nm. The discrepancy between the particle size results and the previous detection results is that the results obtained under the TEM are for the dried nanoparticles. The particle size instrument detects the hydrated particle size (DLS). The hydrodynamic size given by the DLS corresponds to the micelle core, and the expanded corona corresponds to the core size of the micelles. In contrast, TEM usually gives the core size of the micelles in the dry state because the corona is not visible at low electron density. Therefore, the particle size measured under TEM is smaller than that of the particle sizer.

#### 3.1.7. Differential Scanning Calorimetry

**Figure 10:**
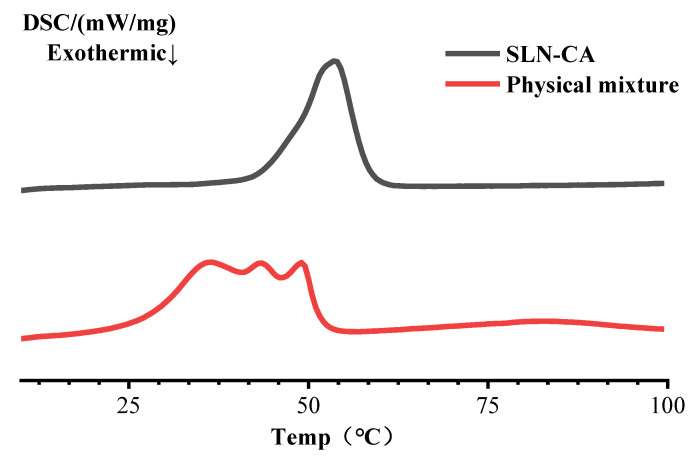
DSC scan results of SLN-CA with the physical mixture.

DSC is a common means of thermal analysis of inclusion compounds. DSC scan results of SLN-CA with the physical mixture (Figure 10). The heat absorption peak in DSC represents the melting of crystals. The results of the analysis recorded by STARe show that the physical mixture starts at 27.5 °C at the beginning of the heat absorption peak To during the temperature rise of the DSC analyzer. The peak temperature Tp was at 36.2 °C, 43.5 °C and 49.0 °C, respectively. Tc ended at 51.7 °C. The integral ΔH is 58.67 J/g. Three peaks of heat absorption throughout the physical mixture. The starting heat absorption peak of SLN-CA during DSC melting starts at 46.4 °C. Tp peaked at 53.7 °C, and Tc ended at 58.3 °C with an integral ΔH of 39.9 J/g. There is only one heat absorption peak throughout the whole process. It is evident that CA is encapsulated, and the findings in SLN with TG-DTG are consistent. The Tc of SLN-CA was shifted back by 6.6 °C compared with the physical mixture. Maybe the water in the SLN cavity was replaced by CA after encapsulation to improve the stability of SLN-CA, resulting in the shifted Tc. The lower melting point of SLN-CA compared to that of Monostearin may be due to two factors: 1. According to the Kelvin effect, the particles are small, the surface area is large, the specific surface Gibbs free energy is more considerable, the chemical potential is higher, it is easier to melt, so the melting point is lower [28,29]. 2. The presence of surfactants and essential oils [30].

#### 3.1.8. Encapsulation Percentage

EN is the percentage of drug entrapped in the nanocarrier relative to the total amount of drug added to the formulation. An excellent solid lipid nanoparticle should have a high EN, and the SLN-CA encapsulation rate prepared in this study was 89.493%.

#### 3.1.9. Determination of Slow Release and Retention

**Figure 11:**
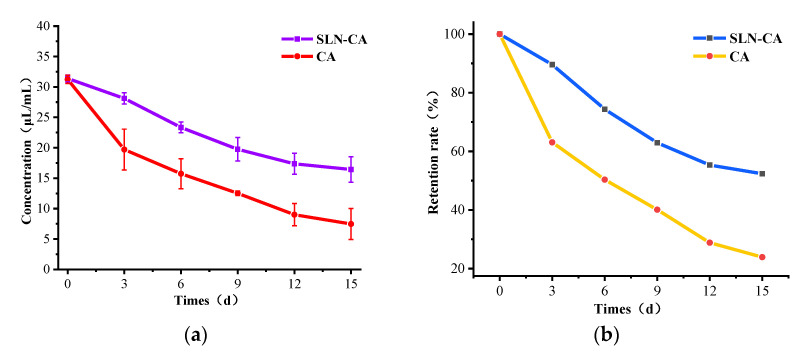
Results of concentration change (**a**) and retention (**b**) of SLN-CA with the same concentration of unencapsulated CA placed at 25 °C for 15 days. Error bars show the standard deviation of the means (three replications).

Sustained release performance and retention are critical indicators to evaluate a product with long-lasting function. The results of the slow-release property and retention ability measurement showed that SLN-CAs with a slow-release function could retain CA well for a long time to function. The concentration of both encapsulated and unencapsulated CA started to decrease from the first day of the experiment. However, the encapsulated product decreased more slowly than the pure CA, with SLN-CA decreasing by 14.95 μL/mL and pure CA decreasing by 23.765 μL/mL after 15 days (Figure 11a). The results showed that after 15 days, SLN-CA retained 52.36% of the essential oil concentration of 15 days prior, an increase of 28.44% retention rate compared with CA (Figure 11b). That is due to the slow release of CA being successfully encapsulated inside the nanoparticles. The slow release and retention experiments further supported the success of the SLN-CA preparation.

#### 3.1.10. Stability

**Figure 12:**
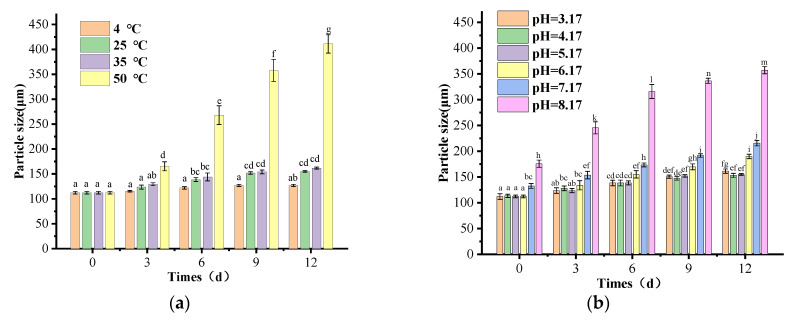
Changes in particle size of SLN-CA at different pH (**b**) and temperature (**a**) for 12 days. Error bars show the standard deviation of the means (three replications); different letters a–m in the graph indicate significant differences between treatments based on Duncan’s multiple range test (*p* < 0.05).

Good stability is essential for a product to be promoted on a large scale. Nanoparticles may change in size and shape during storage [31]. Therefore, we tested the particle size variation of SLN-CA under different storage conditions to investigate its stability capabilities. The particle size increased by 15 ± 2.1 nm, 43 ± 1.6 nm, 49 ± 2.5 nm, and 299 ± 18.3 nm when placed at pH 5.17 for 12 days at 4 °C, 25 °C, 35 °C, 50 °C, and 60 °C, respectively (Figure 12a). Since the sample stratified the next day at 60 °C, it was judged as unstable and unusable without detecting the particle size. The particle size increased by 49 ± 4.1 nm, 40 ± 3.8 nm, 43 ± 1.6 nm, 78 ± 4.3 nm,83 ± 5.2 nm,82 ± 6.9 nm at 25 °C and pH 3.17 to 8.17 storage for 12 days, respectively (Figure 12b). It can be seen that SLN-CA has good stability at 35 °C and below, and is best at 4 °C. It can be seen that SLN-CA is stable at 35 °C and below, and is best at 4 °C. The nanoparticles are suitable for application and storage in acidic environments and have excellent stability [32]. The increase in particle size due to the rise in pH may be due to the disruption of the nanoparticle structure due to the MG saponification reaction [33,34].

### 3.2. Inhibition of Bacteria and Fungi

**Figure 13:**
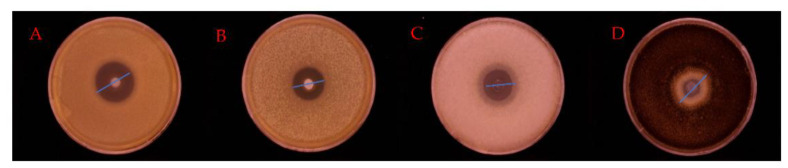
Inhibition circle size of different strains treated with SLN-CA; (**A**) for *Staphylococcus aureus*. (**B**) *Escherichia coli*. (**C**) *Creeping Rhizopus*. (**D**) *Aspergillus niger*.

The inhibition ability of SLN-CA was determined by the punching method, and it can be seen from Figure 13 that SLN-CA has a growth inhibition effect on *Escherichia coli*, *Staphylococcus aureus*, *Creeping Rhizopus*, and *Aspergillus niger*. The results of the diameter of the inhibition circle are shown in Table 2, and the results show that the size of the inhibition circle of *Staphylococcus aureus* is 23.10 ± 0.78 mm, *Escherichia coli* is 21.48 ± 0.44 mm, *Creeping Rhizopus* is 20.66 ± 1.21 mm and *Aspergillus niger* is 18.63 ± 0.95 mm. According to the scoring criteria in Section 2.4., SLN-CA is highly sensitive to *S. aureus*, *E. coli*, and *Rhizopus*, and moderately sensitive to *Aspergillus niger.* It is proven that CA retains its excellent antibacterial ability after being encapsulated into SLN-CA by SLN [35].

## 4. Conclusions

This study reports the successful preparation of SLN-CA along with the simultaneous characterization of nanoparticles using different technical means, and the simultaneous determination of sustained release capacity. The analytical results show that the prepared SLN-CA has high levels of dispersibility and stability. It can be stored stably below 50 °C and under an acidic environment. The overall structure of the nanoparticles was rounded and full under electron microscopy. After the inhibition circle experiment, the results showed that SLN-CA had considerable inhibition ability against *Staphylococcus aureus*, *Escherichia coli.*, *Creeping Rhizopus*, and *Aspergillus niger*.

## Figures and Tables

**Table 1:** Results of particle size, zeta potential, and polydispersity coefficient.

	Particle Size (nm)	Zeta-Potential (mV)	PDI
SLN-CA	74 ± 5	−44.36 ± 2.2	0.153 ± 0.032

Data represent mean ± standard deviation (n = 3).

**Table 2:** SLN-CA antibacterial ability.

Strain	*S. aureus*	*E. coli*	*Rhizopus*	*Aspergillus niger*
Diameter of inhibition circle (mm)	23.10 ± 1.22	21.48 ± 0.85	20.66 ± 3.37	18.63 ± 2.45

Data represent mean ± standard deviation (n = 3).

## Data Availability

The data presented in this study are contained within the article.
